# Causal Information Rate

**DOI:** 10.3390/e23081087

**Published:** 2021-08-21

**Authors:** Eun-jin Kim, Adrian-Josue Guel-Cortez

**Affiliations:** Center for Fluid and Complex Systems, Coventry University, Priory St., Coventry CV1 5FB, UK; guelcortea@uni.coventry.ac.uk

**Keywords:** information geometry, information length, information rate, causality, abrupt events, entropy

## Abstract

Information processing is common in complex systems, and information geometric theory provides a useful tool to elucidate the characteristics of non-equilibrium processes, such as rare, extreme events, from the perspective of geometry. In particular, their time-evolutions can be viewed by the rate (information rate) at which new information is revealed (a new statistical state is accessed). In this paper, we extend this concept and develop a new information-geometric measure of causality by calculating the effect of one variable on the information rate of the other variable. We apply the proposed causal information rate to the Kramers equation and compare it with the entropy-based causality measure (information flow). Overall, the causal information rate is a sensitive method for identifying causal relations.

## 1. Introduction

Entropy-related concepts and information theory [1,2,3,4,5,6,7,8,9] are useful for understanding complex dynamics in equilibrium and out of equilibrium. Examples include information (Shannon) entropy (measuring disorder, or lack of information) [1], Fisher information [2], relative entropy [3], mutual information [10], and their microscopic versions (e.g., trajectory entropy) [11,12], etc. In particular, while, in equilibrium, the Shannon entropy has a unique thermodynamic meaning, this is no longer the case in non-equilibrium, with different proposals for generalized entropies (e.g., see the review paper of Reference [13] and references therein). Recent years have witnessed the increased awareness of information as a useful physical concept, for instance, in resolving the famous Maxwell’s demon paradox [14], setting various thermodynamic inequality/uncertainty relations [15,16,17], and establishing theoretical and conceptual links between physics and biology [18]. Information-related ideas are also useful to uncover unexpected relations between apparently unrelated problems, for instance, the connections between Fisher information and Schrödinger equation, inspiring new development in non-equilibrium statistical mechanics [19].

We have recently proposed information-geometric theory as a powerful tool to understand non-equilibrium stochastic processes that often involve high temporal variabilities and large fluctuations [20,21,22,23,24,25,26,27,28,29,30,31,32], as often the case of rare, extreme events. This is based on the surprisal rate, $r\left(x,t\right)=\partial_{t}s\left(x,t\right)=-\partial_{t}\ln p(x,t)$, where p(x,t) is a probability density function (PDF) of a random variable *x* at time *t*, and s(x,t)=−lnp(x,t) is a local entropy. r(x,t), informing how rapidly p(x,t) or r(x,t) changes in time, is especially useful for understanding time-varying non-equilibrium processes. As the name indicates, the surprisal rate *r* measures the degree of surprise when p(x,t) changes in time (no surprise in equilibrium with r=0). We can easily show that the average of the surprisal rate ∫dxp(x,t)r(x,t)=0 since ∫dxp(x,t)=1. We note that, in this paper, averages refer to ensemble averages, which vary with time. A non-zero value is obtained from the second moment of r(x,t) as

(1) $$E\left(t\right)=\Gamma^{2}\left(t\right)=\int dxp\left(x,t\right){\left(r\left(x,t\right)\right)}^{2}=\int dxp\left(x,t\right){\left(\partial_{t}\ln p(x,t)\right)}^{2},$$

where Γ(t) represents the information rate at which a new information is revealed (a new statistical state is accessed) due to time-evolution. Alternatively, $\tau \left(t\right)=\Gamma {\left(t\right)}^{-1}$ is the characteristic time scale over which information changes, linked to the smallest time scale of fluctuations [17].

It is important to highlight that E, Γ, and τ have the dimensions of (time)${}^{-2}$, (time)${}^{-1}$, and (time), respectively. In addition, we note that E is proportional to the average of an infinitesimal relative entropy (Kullback–Leibler divergence) (e.g., see Reference [20]),

(2) $$\begin{array}{ccc} E & = & \lim_{dt\to 0}\frac{2}{{\left(dt\right)}^{2}}\int dxp\left(x,t+dt\right)\ln\left[\frac{p(x,t+dt)}{p(x,t)}\right]=\lim_{dt\to 0}\frac{2}{{\left(dt\right)}^{2}}\int dxp\left(x,t\right)\ln\left[\frac{p(x,t)}{p(x,t+dt)}\right]. \end{array}$$

The total change in information between the initial time 0 and the time *t* is then obtained by integrating $\frac{1}{\tau (t)}$ over time as $L\left(t\right)=\int_{0}^{t}\frac{dt_{1}}{\tau (t_{1})}$ which is the information length, quantifying the total number of statistically different states that a system passes through in time. In the limit of a Gaussian PDF where the variance is constant in time, one statistically distinguishable state is generated when a PDF peak moves by one standard deviation since the latter provides the uncertainty in measuring the peak position of the PDF. In a nutshell, L is an information-geometric measure, enabling us to quantify how the “information” unfolds in time by dimensionless distance. Unlike other information measures, E, τ, and L are invariant under (time-independent) change of variables and are not system-specific. This non-system-specificity is especially useful for comparing the evolution of different variables/systems having different units.

Furthermore, L is a path-dependent dimensionless distance and is uniquely defined as a function of time for fixed parameters and initial condition. These properties are advantageous for quantifying correlation in time-varying data and understanding self-organization, long memory, and hysteresis involved in phase transitions [20,24,27,28,29,30,32]. In particular, we recently investigated a non-autonomous Kramer equation by including a sudden perturbation to the system to mimic the onset of a sudden event [32], demonstrating that our information rate predicts the onset of a sudden event better than one of the entropy-based measures (information flow) (see Section 5.4 for details).

The purpose of this paper is to develop an information-geometric measure of causality (the causal information rate) by generalizing E (Γ). Like Reference [32], our intention here is not on modeling the appearance of rare, extreme events (that are nonlinear, non-Gaussian) themselves, but on developing a new information-geometric causality method which is useful for predicting and understanding those events. The remainder of this paper is organized as follows. We propose the causal information rate in Section 2 and apply it to the Kramers equation in Section 3. One of the entropy-based methods (the information flow) is calculated in Section 4 and is compared with our proposed method in Section 5. Conclusions are provided in Section 6. Appendix A, Appendix B and Appendix C show some detailed steps involved in our calculations. We note that, while the usual convention in statistics and probability theory is to use upper case letters for random variables and lower case letters for their realizations, we do not make such distinctions in this paper as their meanings should be clear from the context.

## 2. Causal Information Rate

Information theoretical measures of causality are often based on entropy, joint entropy, conditional entropy, or mutual information, where the causality is measured by the improvement of the predictability of one of the variables at future time by the knowledge of the other variable, the improved predictability being measured by the decrease in entropy [16,33,34,35,36,37,38,39,40,41]. However, there have been questions raised as to whether predictability improvement (e.g., as measured by the Granger causality, transfer entropy) is directly linked to causality (e.g., Reference [39]) and the suggestion that causality is better understood by performing an intervention experiment to measure the effect of the change (some type of perturbation or intervention) in one variable on another. In particular, spurious causalities between the two (observed) state variables can arise through unobserved state variables that interact with both (observed) state variables, calling for care in dealing with a system with more than two variables. On the other hand, to deal with strongly time-dependent data, the concepts of transfer entropy rate [34], information flow [16,40,41], etc., are proposed.

It is not the aim of this paper to provide detailed discussions about these methods, but to introduce a new information geometric measure of causality (see below) and to compare our new method with one of them (information flow) (see Section 4). Our new information geometric measure of causality focuses on how one variable affects the information rate of another variable. To this end, we generalize Γ in Equation (1) and define the causal information rate for multiple variables.

In order to demonstrate the basic idea required, it is instructive to consider a stochastic system consisting of two variables $X_{1}$ and $X_{2}$ which have a bivariate joint PDF $p(X_{1},t_{1};X_{2},t_{2})$ at different times $t_{1}$ and $t_{2}$, its equal-time joint PDF $p\left(X_{1},t;X_{2},t\right)\equiv p\left(X_{1},X_{2},t\right)$, and the conditional PDFs $p\left(X_{2},t_{2}|X_{1},t_{1}\right)=p\left(X_{2},t_{2};X_{1},t_{1}\right)/p\left(X_{1},t_{1}\right)$, as well as marginal PDFs $p\left(X_{1},t\right)=\int dX_{2}p\left(X_{1},X_{2},t\right)$ and $p\left(X_{2},t\right)=\int dx_{1}p\left(X_{1},X_{2},t\right)$. Using the index notation i,j=1,2, we then define causal information rate $\Gamma_{i\to j}$ for i≠j from the variable $X_{i}$ to $X_{j}$ as follows:

(3) $$\begin{array}{ccc} \Gamma_{i\to j} & \equiv & \Gamma_{j}^{*}-\Gamma_{j}, \end{array}$$

(4) $$\begin{array}{ccc} E_{j} & \equiv & \Gamma_{j}{\left(t\right)}^{2}=\int dX_{j}\;p\left(X_{j},t\right){\left(\partial_{t}\ln(p\left(X_{j},t\right)\right)}^{2}, \end{array}$$

(5) $$\begin{array}{ccc} E_{j}^{*} & \equiv & \Gamma_{j}^{*}{\left(t\right)}^{2}=\lim_{t_{1}\to t^{+}}\int dX_{i}dX_{j}\;p\left(X_{j},t_{1};X_{i},t\right){\left(\partial_{t_{1}}\ln\left[p\left(X_{j},t_{1}|X_{i},t\right)\right]\right)}^{2}\\ & = & \lim_{t_{1}\to t^{+}}\int dX_{i}dX_{j}\;p\left(X_{j},t_{1};X_{i},t\right){\left(\partial_{t_{1}}\ln\left[p\left(X_{j},t_{1};X_{i},t\right)\right]\right)}^{2}. \end{array}$$

Here, $\partial_{t_{1}}p\left(X_{i},t\right)=0$ for $t_{1}\neq t$ was used. $\Gamma_{i}=\frac{1}{\tau_{i}\left(t\right)}$ represents the information rate of $X_{i}$ with its characteristic timescale $\tau_{i}\left(t\right)$. Note that the subscript *j* in $\Gamma_{j}$ denotes that the information rate is calculated for the variable *j*. Since $\Gamma_{j}$ contains the contribution from the variable *j* itself and other variable i≠j, we denote the (auto) contribution from *j*-th itself (where the other variable i≠j is frozen in time) by using the superscript *. That is, $\Gamma_{j}^{*}$ represents the information rate of $X_{j}$ for given (frozen) $X_{i}$. Subtracting $\Gamma_{j}$ from $\Gamma_{j}^{*}$ in Equation (3) then gives us the contribution of dynamic (time-evolving) $X_{i}$ to $\Gamma_{j}$, signifying how $X_{i}$ instantaneously influences the information rate of $X_{j}$.

It is important to note that, as in the case of the information rate Γ or L, the calculation of $\Gamma_{i\to j}$, $\Gamma_{j}$ and $\Gamma_{j}^{*}$ in Equations (3)–(5) does not require the knowledge of the main governing equations (stochastic differential equations). This is because Equations (3)–(5) can be calculated from any (numerical or experimental) data as long as time-dependent (marginal, joint) PDFs can be constructed. For instance, we used a time-sliding window method to construct time-dependent PDFs of different variables and then calculated E and L to analyze numerically generated time-series data for fusion turbulence [26], time-series music data [20], and numerically generated time-series data for global circulation model [28]. However, it is not always clear how many hidden variables are in a given data set.

It is also useful to note that, as in the case of Equation (2), Equation (5) can be shown to be related to the infinitesimal relative entropy as

(6) $$\begin{array}{ccc} E_{j}^{*} & = & {\Gamma_{j}^{*}}^{2}=2\lim_{dt\to 0}\frac{1}{{\left(dt\right)}^{2}}\int dX_{i}dX_{j}\;p\left(X_{j},t+dt;X_{i},t\right)\ln\left[\frac{p(X_{j},t+dt;X_{i},t)}{p(X_{j},X_{i},t)}\right]. \end{array}$$

The method presented above is for a stochastic process with two variables. For stochastic processes involving three or more variables (i,j=1,2,…n, n≥3), one way to proceed is to calculate multivariate PDFs, and then bivariate joint PDFs $p(X_{j},t_{1};X_{j},t_{2})$ and its equal-time joint PDF $p\left(X_{i},t;X_{j},t\right)\equiv p\left(X_{i},X_{j},t\right)$, and marginal PDFs $p(X_{i},t)$ and $p(X_{j},t)$, and then calculate the information rate from $X_{i}$ to $X_{j}$, where i≠j (i,j=1,2,…n, n≥3), via Equations (3)–(5). This will give us an effective causal information rate. Another way is to deal with the multivariate PDFs directly (to be reported in future work).

## 3. Kramers Equation

To demonstrate how the methods Equations (3)–(5) work, in this section, we investigate an analytically solvable, Kramers equation, governed by the following Langevin equations [31,42]:

(7) $$\begin{array}{ccc} \frac{dx}{dt} & = & v, \end{array}$$

(8) $$\begin{array}{ccc} \frac{dv}{dt} & = & -\gamma v-\omega^{2}x+\xi . \end{array}$$

Here, ξ is a short (delta) correlated Gaussian noise with a zero average (mean) 〈ξ〉=0 and the strength *D* with the following property:

(9) $$\langle \xi \left(t\right)\xi \left(t^{\prime }\right)\rangle =2D\delta \left(t-t^{\prime }\right),$$

where the angular brackets denote the average over ξ (〈ξ〉=0).

Assuming an initial Gaussian PDF, time-dependent PDFs remain Gaussian for all time. Thus, the bivariate joint PDF $p(x,t_{1};v,t_{2})$ and the marginal PDFs p(x,t) and p(v,t) are completely determined by covariance and mean values as

(10) $$\begin{array}{ccc} p(x,t_{1};v,t_{2}) & = & \frac{1}{\left(2\pi \right)\sqrt{\left|\Sigma (t_{1},t_{2})\right|}}\exp\left(-\frac{1}{2}\Sigma_{ij}^{-1}\left(t_{1},t_{2}\right)\left(X_{i}-\langle X_{i}\rangle \right)\left(X_{j}-\langle X_{j}\rangle \right)\right), \end{array}$$

(11) $$\begin{array}{ccc} p(x,t) & = & \sqrt{\frac{\beta_{x}}{\pi }}\exp\left(-\beta_{x}{\left(x-\langle x\left(t\right)\rangle \right)}^{2}\right), \end{array}$$

(12) $$\begin{array}{ccc} p(v,t) & = & \sqrt{\frac{\beta_{v}}{\pi }}\exp\left(-\beta_{v}{\left(v-\langle v\left(t\right)\rangle \right)}^{2}\right). \end{array}$$

Here, $\left(X_{1},X_{2}\right)=\left(x\left(t_{1}\right),v\left(t_{2}\right)\right)$. 〈x〉 and 〈v〉 are the mean values. $\Sigma (t_{1},t_{2})$ is the covariance matrix with the elements $\Sigma_{11}=\Sigma_{xx}\left(t_{1}\right)=\langle {\left(\delta x\left(t_{1}\right)\right)}^{2}\rangle =\frac{1}{2\beta_{x}\left(t_{1}\right)}$, $\Sigma_{12}=\Sigma_{21}=\Sigma_{xv}\left(t_{1},t_{2}\right)=\langle \delta x\left(t_{1}\right)\delta v\left(t_{2}\right)\rangle$, and $\Sigma_{22}=\Sigma_{vv}\left(t_{2}\right)=\langle {\left(\delta v\left(t_{2}\right)\right)}^{2}\rangle =\frac{1}{2\beta_{v}\left(t_{2}\right)}$, where $\delta x\left(t_{1}\right)=x-\langle x\left(t_{1}\right)\rangle$ and $\delta v\left(t_{2}\right)=v\left(t_{2}\right)-\langle v\left(t_{2}\right)\rangle$. $\Sigma^{-1}$ is the inverse of Σ, while $\left|\Sigma \right|=\Sigma_{11}\Sigma_{22}-\Sigma_{12}^{2}$ is the determinant. Appendix A shows how to calculate mean values and the elements of covariance matrix.

Entropy of the joint PDF $p(x,t_{1};v,t_{2})$ and marginal PDFs p(x,t) and p(v,t) can easily be shown to be

(13) $$\begin{array}{ccc} S(t_{1},t_{2}) & = & -\int dxdv\;p\left(x,t_{1};v,t_{2}\right)\ln p(x,t_{1};v,t_{2})=\frac{1}{2}\left[1+\ln\left({\left(2\pi \right)}^{2}|\Sigma \left(t_{1},t_{2}\right)\right|)\right], \end{array}$$

(14) $$\begin{array}{ccc} S_{x}\left(t\right) & = & -\int dxp\left(x,t\right)\ln p(x,t)=\frac{1}{2}\left[1+\ln(2\pi \Sigma_{xx}\left(t\right))\right], \end{array}$$

(15) $$\begin{array}{ccc} S_{v}\left(t\right) & = & -\int dvp\left(v,t\right)\ln p(v,t)=\frac{1}{2}\left[1+\ln(2\pi \Sigma_{vv}\left(t\right))\right]. \end{array}$$

On the other hand, the information rates for the equal-time joint PDF and the marginal PDFs are given by

(16) $$\begin{array}{ccc} E & = & \Gamma^{2}=\int dxdvp\left(x,v,t\right){\left(\partial_{t}\ln p(x,v,t)\right)}^{2}=\partial_{t}\langle X_{i}\rangle \Sigma_{ij}^{-1}\partial_{t}\langle X_{j}\rangle +\frac{1}{2}\mathrm{Tr}\left[{\left(\Sigma^{-1}\dot{\Sigma }\right)}^{2}\right], \end{array}$$

(17) $$\begin{array}{ccc} E_{x} & = & \Gamma_{x}^{2}=\int dxp\left(x,t\right){\left(\partial_{t}\ln p(x,t)\right)}^{2}=\frac{1}{\Sigma_{xx}}{\left(\frac{d\langle x\rangle }{dt}\right)}^{2}+\frac{1}{2\Sigma_{xx}^{2}}{\left(\frac{d\Sigma_{xx}}{dt}\right)}^{2}, \end{array}$$

(18) $$\begin{array}{ccc} E_{v} & = & \Gamma_{v}^{2}=\int dvp\left(v,t\right){\left(\partial_{t}\ln p(v,t)\right)}^{2}=\frac{1}{\Sigma_{vv}}{\left(\frac{d\langle v\rangle }{dt}\right)}^{2}+\frac{1}{2\Sigma_{vv}^{2}}{\left(\frac{d\Sigma_{vv}}{dt}\right)}^{2}. \end{array}$$

It is useful to note that the first term on the RHS of Equations (17) and (18) is caused by the temporal change in the mean values of *x* and *v*, respectively, while the second term is due to that in the variance. Equation (16) for the joint PDF contains the contribution from the temporal changes in the mean values of *x* and *v* and in the covariance matrix. The derivation of Equations (16)–(18) is provided in Appendix B (also see Reference [31]).

To clarify the key idea behind the causality information rate, we provide detailed mathematical steps involved in the definition and calculation of $\Gamma_{x\to v}$ and $\Gamma_{v\to x}$ in Section 3.1 and Section 3.2, respectively.

### 3.1. $\Gamma_{v\to x}$

We start with the Kramers process Equations (7)–(9), where $X_{2}=v$ is frozen for time $\left(t,t_{1}\right)$;

(19) $$\begin{array}{ccc} \frac{dx}{dt} & = & v, \end{array}$$

(20) $$\begin{array}{ccc} \frac{dv}{dt} & = & 0. \end{array}$$

Then, the bivariate Gaussian PDF in Equation (10) for a fixed *v* takes the following form:

(21) $$\begin{array}{ccc} & & p\left(x,t_{1};v,t\right)=\frac{1}{\left(2\pi \right)\sqrt{\left|\Sigma (t_{1},t)\right|}}\exp\left(-\frac{1}{2}\Sigma_{ij}^{-1}\left(t_{1},t\right)\left(X_{i}-\langle X_{i}\rangle \right)\left(X_{j}-\langle X_{j}\rangle \right)\right), \end{array}$$

(22) $$\begin{array}{ccc} & & \Sigma \left(t_{1},t\right)=\left[\begin{array}{cc} \Sigma_{xx}\left(t_{1}\right) & \Sigma_{xv}\left(t_{1},t\right)\\ \Sigma_{xv}\left(t_{1},t\right) & \Sigma_{vv}\left(t\right) \end{array}\right]=\left[\begin{array}{cc} \langle {\left(\delta x\left(t_{1}\right)\right)}^{2}\rangle & \langle \delta x\left(t_{1}\right)\delta v\left(t\right)\rangle \\ \langle \delta x\left(t_{1}\right)\delta v\left(t\right)\rangle & \langle {\left(\delta v\left(t\right)\right)}^{2}\rangle \end{array}\right], \end{array}$$

where $X_{1}=x\left(t_{1}\right)$ and $X_{2}\left(t_{2}\right)$, $\langle X_{1}\rangle =\langle x\left(t_{1}\right)\rangle$, $\langle X_{2}\rangle =\langle v\left(t\right)\rangle$, $\delta x\left(t_{1}\right)=x\left(t_{1}\right)-\langle x\left(t_{1}\right)\rangle$, and δv(t)=v(t)−〈v(t)〉.

For i=2 and j=1 in Equations (3) and (5), we have

(23) $$\begin{array}{ccc} \Gamma_{v\to x}\left(t\right) & = & \Gamma_{x}^{*}-\Gamma_{x}, \end{array}$$

(24) $$\begin{array}{ccc} E_{x}^{*}\left(t\right) & = & \Gamma_{x}^{*}{\left(t\right)}^{2}=\lim_{t_{1}\to t^{+}}\int dxdv\;p\left(x,t_{1};v,t\right){\left(\partial_{t_{1}}\ln p(x,t_{1};v,t)\right)}^{2}, \end{array}$$

where $\Gamma_{x}=\sqrt{E_{x}}$. In Appendix B, we show that $E_{x}^{*}$ is given by

(25) $$\begin{array}{ccc} E_{x}^{*} & = & \lim_{t_{1}\to t^{+}}\left[\partial_{t_{1}}\langle X_{i}\rangle \Sigma_{ij}^{-1}\left(t_{1},t\right)\partial_{t_{1}}\langle X_{j}\rangle +\frac{1}{2}\mathrm{Tr}\left[{\left(\Sigma^{-1}\left(t_{1},t\right)\partial_{t_{1}}\Sigma \left(t_{1},t\right)\right)}^{2}\right]\right]. \end{array}$$

Since *v* is frozen during time $\left(t,t_{1}\right)$, $\lim_{t_{1}\to t}\partial_{t_{1}}\langle X_{i}\rangle =\delta_{i1}\partial_{t}\langle x\left(t\right)\rangle$; $\Sigma_{vv}$ remains constant, while $\Sigma_{xx}$ and $\Sigma_{xv}$ change, as follows:

(26) $$\begin{array}{ccc} \lim_{t_{1}\to t}\partial_{t_{1}}\Sigma_{vv}\left(t\right) & = & 0, \end{array}$$

(27) $$\begin{array}{ccc} \lim_{t_{1}\to t}\partial_{t_{1}}\Sigma_{xv}\left(t_{1},t\right) & = & \lim_{t_{1}\to t}\langle \partial_{t_{1}}\left(\delta x\left(t_{1}\right)\right)\delta v\left(t\right)\rangle =\Sigma_{vv}\left(t\right)\equiv {\dot{\Sigma }}_{xv}, \end{array}$$

(28) $$\begin{array}{ccc} \lim_{t_{1}\to t}\partial_{t_{1}}\Sigma_{xx}\left(t_{1}\right) & = & \lim_{t_{1}\to t}\langle \partial_{t_{1}}\left(\delta x\left(t_{1}\right)\right)\delta x\left(t_{1}\right)\rangle =2\Sigma_{vx}\left(t\right)\equiv {\dot{\Sigma }}_{xx}. \end{array}$$

Then, to calculate the two terms on RHS of Equation (25), we note

(29) $$\begin{array}{ccc} \Sigma^{-1}\left(t_{1},t\right)=\frac{1}{\left|\Sigma \right|}\left[\begin{array}{cc} \Sigma_{vv}\left(t\right) & -\Sigma_{xv}\left(t_{1},t\right)\\ -\Sigma_{xv}\left(t_{1},t\right) & \Sigma_{xx}\left(t\right) \end{array}\right], & & \lim_{t_{1}\to t}\partial_{t_{1}}\Sigma \left(t_{1},t\right)=\left[\begin{array}{cc} {\dot{\Sigma }}_{xx} & {\dot{\Sigma }}_{xv}\\ {\dot{\Sigma }}_{xv} & 0 \end{array}\right], \end{array}$$

where ${\dot{\Sigma }}_{xx}$ and ${\dot{\Sigma }}_{xv}$ are given in Equations (27) and (28), while $\Sigma^{-1}=\Sigma^{-1}\left(t\right)$ is the inverse of the equal time covariance matrix. Using Equation (29), we can show that the second term on RHS of Equation (25) becomes

(30) $$\begin{array}{ccc} & & \lim_{t_{1}\to t^{+}}\left[\frac{1}{2}\mathrm{Tr}\left[{\left(\Sigma^{-1}\left(t_{1},t\right)\partial_{t_{1}}\Sigma \left(t_{1},t\right)\right)}^{2}\right]\right]\\ & & =\frac{1}{{2|\Sigma |}^{2}}\left[{\left(\Sigma_{vv}{\dot{\Sigma }}_{xx}-\Sigma_{xv}{\dot{\Sigma }}_{xv}\right)}^{2}+2\left(\Sigma_{vv}{\dot{\Sigma }}_{xv}\right)\left(-\Sigma_{xv}{\dot{\Sigma }}_{xx}+\Sigma_{xx}{\dot{\Sigma }}_{xv}\right)+{\left(\Sigma_{xv}{\dot{\Sigma }}_{xv}\right)}^{2}\right]\\ & & =\frac{1}{{2|\Sigma |}^{2}}\left[2|\Sigma |{\dot{\Sigma }}_{xv}^{2}+{\left(\Delta_{vx}\right)}^{2}\right]. \end{array}$$

Here,

(31) $$\begin{array}{ccc} \Delta_{vx} & \equiv & \Sigma_{vv}{\dot{\Sigma }}_{xx}-2{\dot{\Sigma }}_{xv}\Sigma_{xv}=2\Sigma_{vv}\Sigma_{xv}-2\Sigma_{vv}\Sigma_{xv}=0, \end{array}$$

where ${\dot{\Sigma }}_{xx}=2\Sigma_{xv}$ and ${\dot{\Sigma }}_{xv}=\Sigma_{vv}$ in Equations (27) and (28) are used. It is useful to note that $\Delta_{vx}$ represents the rate at which the determinant of the covariance matrix changes in time for a fixed *v* and becomes zero. This is because, for a fixed *v* (essentially for γ=D=0 as seen below in regard to Equation (52)), the evolution is conservative (reversible) where the phase space volume is conserved. Thus, the contribution from the variance to the information rate of *x* for a given *v* is solely determined by the temporal change in the cross-correlation $\Sigma_{xv}$.

Finally, by using Equations (30) and (31) in Equation (25), we have

(32) $$\begin{array}{ccc} E_{x}^{*} & = & \Sigma_{xx}^{-1}{\langle v\left(t\right)\rangle }^{2}+\frac{{\dot{\Sigma }}_{xv}^{2}}{\left|\Sigma \right|}. \end{array}$$

It is interesting to compare the first term (caused by the mean motion $\frac{d\langle x\rangle }{dt}$) on the RHS of Equation (32) with that in Equation (17). For instance, if $\Sigma_{xv}=0$, $\Sigma_{xx}^{-1}=\frac{\Sigma_{vv}}{\left|\Sigma \right|}=\frac{1}{\Sigma_{xx}}$, they take the same value. It should also be noted that, even when both $\Sigma_{xv}=0$ and $\frac{d}{dt}\Sigma_{xv}=\Sigma_{vv}-\omega^{2}\Sigma_{xx}=0$ (as in equilibrium), ${\dot{\Sigma }}_{xv}=\Sigma_{vv}\neq 0$ (unless $\Sigma_{vv}=0$).

Putting Equations (17) and (32) and $\lim_{t_{1}\to t}\partial_{t_{1}}\langle X_{i}\rangle =\delta_{i1}\partial_{t}\langle x\left(t\right)\rangle$ in Equation (23) gives us

(33) $$\begin{array}{ccc} \Gamma_{v\to x} & = & \sqrt{E_{x}^{*}}-\sqrt{E_{x}}\\ & = & {\left[\frac{\Sigma_{vv}{\langle v\left(t\right)\rangle }^{2}}{\left|\Sigma \right|}+\frac{\Sigma_{vv}^{2}}{\left|\Sigma \right|}\right]}^{\frac{1}{2}}-{\left[\frac{{\langle v\left(t\right)\rangle }^{2}}{\Sigma_{xx}}+\frac{2\Sigma_{xv}^{2}}{\Sigma_{xx}^{2}}\right]}^{\frac{1}{2}}, \end{array}$$

where we used ${\dot{\Sigma }}_{xv}=\Sigma_{vv}$ and ${\dot{\Sigma }}_{xx}=2\Sigma_{xv}$. (See Appendix A for the values for means and covariance matrix.) Therefore, even when $\Sigma_{xv}=0$, Equation (33) can have a non-trivial contribution from a non-zero mean velocity.

To understand the difference between $E_{x}^{*}$ and $E_{x}$, it is useful to define the following quantify

(34) $$\begin{array}{ccc} E_{v\to x} & = & E_{x}^{*}-E_{x}=\frac{\Sigma_{xv}^{2}}{\left|\Sigma \right|\Sigma_{xx}}{\langle v\left(t\right)\rangle }^{2}+\frac{{\left|\Sigma \right|}^{2}+\Sigma_{xv}^{4}}{\left|\Sigma \right|\Sigma_{xx}^{2}}. \end{array}$$

The cross-correlation $\Sigma_{xv}$ plays a more important role in Equation (34) than in Equation (33). For instance, $\Sigma_{xv}=0$ reduces Equation (34) into a simple form $E_{v\to x}=\frac{\Sigma_{vv}}{\Sigma_{xx}}$, with no contribution from the mean velocity *v*. As noted above, such simplification does not occur for $\Gamma_{v\to x}$ in Equation (33).

Nevertheless, if $\frac{d\langle x\rangle }{dt}=\frac{d\langle v\rangle }{dt}=0$ and $\Sigma_{xv}=0$, Equations (33) and (34) become

(35) $$E_{v\to x}=\frac{\Sigma_{vv}}{\Sigma_{xx}},\;\;\;\;\Gamma_{v\to x}=\sqrt{\frac{\Sigma_{vv}}{\Sigma_{xx}}}.\;\;\;\;$$

For instance, in equilibrium, $\Sigma_{xv}=0$, $\Sigma_{xx}=\frac{D}{\gamma \omega^{2}}$, $\Sigma_{vv}=\frac{D}{\gamma }$; thus, $\Gamma_{v\to x}=\omega$. In Section 3.2 below, we show that the equality $\Gamma_{v\to x}=\Gamma_{x\to v}=\omega$ holds in equilibrium (see the discussion below Equation (57)).

### 3.2. $\Gamma_{x\to v}$

We now consider the Kramers equation Equations (7)–(9), where $X_{1}=x$ is frozen for time $\left(t,t_{1}\right)$;

(36) $$\begin{array}{ccc} \frac{dx}{dt} & = & 0, \end{array}$$

(37) $$\begin{array}{ccc} \frac{dv}{dt} & = & -\gamma v-\omega^{2}x+\xi . \end{array}$$

Then, during $\left(t,t_{1}\right)$, the bivariate Gaussian PDF in Equation (10) for a fixed *x* takes the following form:

(38) $$\begin{array}{ccc} & & p\left(x,t;v,t_{1}\right)=\frac{1}{\left(2\pi \right)\sqrt{\left|\Sigma (t,t_{1})\right|}}\exp\left(-\frac{1}{2}\Sigma_{ij}^{-1}\left(t,t_{1}\right)\left(X_{i}-\langle X_{i}\rangle \right)\left(X_{j}-\langle X_{j}\rangle \right)\right), \end{array}$$

(39) $$\begin{array}{ccc} & & \Sigma \left(t,t_{1}\right)=\left[\begin{array}{cc} \Sigma_{xx}\left(t\right) & \Sigma_{xv}\left(t,t_{1}\right)\\ \Sigma_{xv}\left(t,t_{1}\right) & \Sigma_{vv}\left(t_{1}\right) \end{array}\right]=\left[\begin{array}{cc} \langle {\left(\delta x\left(t\right)\right)}^{2}\rangle & \langle \delta x\left(t\right)\delta v\left(t_{1}\right)\rangle \\ \langle \delta x\left(t\right)\delta v\left(t_{1}\right)\rangle & \langle {\left(\delta v\left(t_{1}\right)\right)}^{2}\rangle \end{array}\right], \end{array}$$

where $\langle X_{1}\rangle =\langle x\left(t\right)\rangle$, $\langle X_{2}\rangle =\langle v\left(t_{1}\right)\rangle$, δx(t)=x(t)−〈x(t)〉, and $\delta v\left(t_{1}\right)=v\left(t_{1}\right)-\langle v\left(t_{1}\right)\rangle$.

We define $E_{v}^{*}$, $\Gamma_{v}^{*}=\sqrt{E_{v}^{*}}$, $\Gamma_{v}=\sqrt{E_{v}}$, and $E_{x\to v}$ as

(40) $$\begin{array}{ccc} \Gamma_{x\to v} & = & \Gamma_{v}^{*}-\Gamma_{v}, \end{array}$$

(41) $$\begin{array}{ccc} E_{x\to v} & = & E_{v}^{*}-E_{v}, \end{array}$$

(42) $$\begin{array}{ccc} E_{v}^{*}\left(t\right) & = & \Gamma_{v}^{*}{\left(t\right)}^{2}=\lim_{t_{1}\to t^{+}}\int dxdvp\left(x,t;v,t_{1}\right){\left(\partial_{t_{1}}\ln p(x,t;v,t_{1})\right)}^{2} \end{array}$$

(43) $$\begin{array}{ccc} & = & \lim_{t_{1}\to t^{+}}\left[\partial_{t_{1}}\langle X_{i}\rangle \Sigma_{ij}^{-1}\left(t,t_{1}\right)\partial_{t_{1}}\langle X_{j}\rangle +\frac{1}{2}\mathrm{Tr}\left[{\left(\Sigma^{-1}\left(t,t_{1}\right)\partial_{t_{1}}\Sigma (t,t_{1})\right)}^{2}\right]\right], \end{array}$$

where $E_{v}$ is given in Equation (18). Note that Equation (43) simply follows by replacing $\Sigma (t_{1},t)$ by $\Sigma (t,t_{1})$ in Equation (25).

Since we are considering the evolution of joint PDF of *v* for a given *x* for an infinitesimal time interval $\left(t,t_{1}\right)$ through Equations (36) and (37), $\Sigma_{xx}$ remains constant, while $\Sigma_{xv}$ and $\Sigma_{vv}$ evolve in time as follows:

(44) $$\begin{array}{ccc} \lim_{t_{1}\to t}\partial_{t_{1}}\Sigma_{xx}\left(t\right) & = & \lim_{t_{1}\to t}\langle \partial_{t_{1}}\left(\delta x\left(t\right)\right)\delta x\left(t\right)\rangle =0, \end{array}$$

(45) $$\begin{array}{ccc} \lim_{t_{1}\to t}\partial_{t_{1}}\Sigma_{xv}\left(t,t_{1}\right) & = & \lim_{t_{1}\to t}\langle \delta x\left(t\right)\partial_{t_{1}}\left(\delta v\left(t_{1}\right)\right)\rangle \equiv {\Sigma^{\prime }}_{xv}, \end{array}$$

(46) $$\begin{array}{ccc} \lim_{t_{1}\to t}\partial_{t_{1}}\Sigma_{vv}\left(t_{1}\right) & = & 2\lim_{t_{1}\to t}\langle \left(\partial_{t_{1}}\delta v\left(t_{1}\right)\right)\delta v\left(t_{1}\right)\rangle \equiv {\Sigma^{\prime }}_{vv}. \end{array}$$

Here, by using Equations (7)–(9), we can show (see Appendix C for comments):

(47) $$\begin{array}{ccc} {\Sigma^{\prime }}_{vv}\left(t\right) & = & 2\langle (\partial_{t}\left(\delta v\left(t\right)\right)\delta v\left(t\right)\rangle =2\langle \left(-\gamma \delta v\left(t\right)-\omega^{2}\delta x\left(t\right)+\xi \right)\delta v\left(t\right)\rangle \\ & = & 2(-\gamma \Sigma_{vv}-\omega^{2}\Sigma_{xv}+D), \end{array}$$

(48) $$\begin{array}{ccc} {\Sigma^{\prime }}_{xv}\left(t\right) & = & \langle (\partial_{t}\left(\delta v\left(t\right)\right)\delta x\left(t\right)\rangle =\langle \left(-\gamma \delta v\left(t\right)-\omega^{2}\delta x\left(t\right)+\xi \right)\delta x\left(t\right)\rangle =-\gamma \Sigma_{xv}-\omega^{2}\Sigma_{xx}. \end{array}$$

We now need to calculate the two terms on RHS of Equation (43). First, since $X_{1}=x\left(t\right)$ is frozen,

(49) $$\begin{array}{ccc} \lim_{t_{1}\to t^{+}}\partial_{t_{1}}\langle X_{i}\rangle \Sigma_{ij}^{-1}\left(t,t_{1}\right)\partial_{t_{1}}\langle X_{j}\rangle & = & \Sigma_{vv}^{-1}\left(t\right){\left(\partial_{t}\langle v\left(t\right)\rangle \right)}^{2}, \end{array}$$

(50) $$\begin{array}{ccc} \lim_{t_{1}\to t^{+}}\partial_{t_{1}}\Sigma \left(t,t_{1}\right) & = & \left[\begin{array}{cc} 0 & {\Sigma^{\prime }}_{xv}\\ {\Sigma^{\prime }}_{xv} & {\Sigma^{\prime }}_{vv} \end{array}\right]. \end{array}$$

Secondly, using Equations (50) and (39), we can show that the second term on RHS of Equation (43) becomes

(51) $$\begin{array}{ccc} & & \lim_{t_{1}\to t^{+}}\left[\frac{1}{2}\mathrm{Tr}\left[{\left(\Sigma^{-1}\left(t,t_{1}\right)\partial_{t_{1}}\Sigma (t,t_{1})\right)}^{2}\right]\right]\\ & & =\frac{1}{{2|\Sigma |}^{2}}\left[{\left(\Sigma_{xx}{\Sigma^{\prime }}_{vv}-\Sigma_{xv}{\Sigma^{\prime }}_{xv}\right)}^{2}+2\left(\Sigma_{xx}{\Sigma^{\prime }}_{xv}\right)\left(-\Sigma_{xv}{\Sigma^{\prime }}_{vv}+\Sigma_{vv}{\Sigma^{\prime }}_{xv}\right)+{\left(\Sigma_{xv}{\Sigma^{\prime }}_{xv}\right)}^{2}\right]\\ & & =\frac{1}{{2|\Sigma |}^{2}}\left[2|\Sigma |{\Sigma^{\prime }}_{xv}^{2}+{\left(\Delta_{vx}\right)}^{2}\right]. \end{array}$$

Here,

(52) $$\begin{array}{ccc} \Delta_{xv} & \equiv & \Sigma_{xx}{\Sigma^{\prime }}_{vv}-2{\Sigma^{\prime }}_{xv}\Sigma_{xv}=-2\gamma \left|\Sigma \right|+2D\Sigma_{xx}, \end{array}$$

where we used Equations (47) and (48). It is useful to note that $\Delta_{xv}$ in Equation (52), representing the rate at which the determinant of the covariance changes in time for a fixed *x*, contains the two terms involving γ (damping) and *D* (stochasticity) due to irreversibility. We also note that, in equilibrium, $\Sigma_{vv}=\frac{D}{\gamma }$ and $\Sigma_{xv}=0$; thus, $\Delta_{xv}=0$; $\frac{d}{dt}\Sigma_{xv}=0$, but ${\Sigma^{\prime }}_{vx}=-\gamma \Sigma_{xv}-\omega^{2}\Sigma_{xx}=-\omega^{2}\Sigma_{xx}\neq 0$ in Equation (51), in general, contributing to $E_{v}^{*}$ in Equation (43).

We use Equations (49), (51) and (52) in Equation (43) to obtain

(53) $$\begin{array}{ccc} E_{v}^{*} & = & \Sigma_{vv}^{-1}{\langle \partial_{t}v\left(t\right)\rangle }^{2}+\frac{1}{{2|\Sigma |}^{2}}\left(2|\Sigma |{\Sigma^{\prime }}_{xv}^{2}+{\left(\Delta_{vx}\right)}^{2}\right), \end{array}$$

where $\Sigma_{vv}^{-1}=\frac{\Sigma_{xx}}{\left|\Sigma \right|}$. Using Equation (53) and (18) in Equation (40) gives us

(54) $$\begin{array}{ccc} \Gamma_{x\to v} & = & \sqrt{E_{x}^{*}}-\sqrt{E_{x}}\\ & = & {\left[\frac{{\langle \partial_{t}v\left(t\right)\rangle }^{2}}{\Sigma_{vv}}+\frac{1}{2\Sigma_{vv}^{2}}{\left(\frac{d\Sigma_{vv}}{dt}\right)}^{2}\right]}^{\frac{1}{2}}-{\left[\frac{\Sigma_{xx}{\langle \partial_{t}v\left(t\right)\rangle }^{2}}{\left|\Sigma \right|}+\frac{2|\Sigma |{\Sigma^{\prime }}_{xv}^{2}+{\left(\Delta_{vx}\right)}^{2}}{{2|\Sigma |}^{2}}\right]}^{\frac{1}{2}}, \end{array}$$

where $\frac{d\Sigma_{vv}}{dt}=2\left(-\gamma \Sigma_{vv}-\omega^{2}\Sigma_{xv}+D\right)$, ${\Sigma^{\prime }}_{xv}=-\gamma \Sigma_{xv}-\omega^{2}\Sigma_{xx}$ and $\Delta_{vx}=-2\gamma \left|\Sigma \right|+2D\Sigma_{xx}$.

Again, to understand the difference between $E_{x}$ and $E_{x}^{*}$, we perform straightforward but lengthy calculations using Equations (18), (41), (52), and (53) and find the following:

(55) $$\begin{array}{ccc} E_{x\to v} & = & \Sigma_{vv}^{-1}{\langle \partial_{t}v\left(t\right)\rangle }^{2}+\frac{1}{{2|\Sigma |}^{2}}\left[2|\Sigma |{\Sigma^{\prime }}_{xv}^{2}+{\left(\Delta_{vx}\right)}^{2}\right]-\left[\frac{{\langle \partial_{t}v\left(t\right)\rangle }^{2}}{\Sigma_{vv}}+\frac{{\Sigma^{\prime }}_{vv}^{2}}{2\Sigma_{vv}^{2}}\right]\\ & \equiv & \frac{\Sigma_{xv}^{2}}{\left|\Sigma \right|\Sigma_{vv}}{\langle \partial_{t}v\left(t\right)\rangle }^{2}+Q(t). \end{array}$$

Here, *Q* is defined by

(56) $$\begin{array}{ccc} Q & = & \frac{1}{{2|\Sigma |}^{2}}\left[2|\Sigma |{\Sigma^{\prime }}_{xv}^{2}+{\left(\Delta_{vx}\right)}^{2}\right]-\frac{{\Sigma^{\prime }}_{vv}^{2}}{2\Sigma_{vv}^{2}}\\ & = & \frac{1}{\left|\Sigma \right|\Sigma_{vv}^{2}}\left[(2D\Sigma_{xv}-\gamma \Sigma_{xv}\Sigma_{vv}+\left|\Sigma \right|\omega^{2}){}^{2}+2\gamma \omega^{2}\Sigma_{xv}^{3}\Sigma_{vv}+\Sigma_{xv}^{4}\omega^{4}+2D^{2}\frac{\Sigma_{xv}^{4}}{\left|\Sigma \right|}\right]. \end{array}$$

We again note that $\Sigma_{xv}$ plays a key role in Equation (55), as in the case of Equation (34). In particular, if $\Sigma_{xv}=0$, $Q=\frac{\left|\Sigma \right|\omega^{4}}{\Sigma_{vv}^{2}}$. If both $\frac{d\langle x\rangle }{dt}=\frac{d\langle v\rangle }{dt}=0$ and $\Sigma_{xv}=0$, Equations (40) and (55) become

(57) $$\Gamma_{x\to v}=\omega^{2}\sqrt{\frac{\Sigma_{xx}}{\Sigma_{vv}}},\;\;\;\;E_{x\to v}=\omega^{4}\frac{\Sigma_{xx}}{\Sigma_{vv}}.\;\;\;\;$$

In equilibrium where $\Sigma_{xx}=\frac{D}{\gamma \omega^{2}}$ and $\Sigma_{vv}=\frac{D}{\gamma }$, $\Gamma_{x\to v}=\omega$ and $E_{x\to v}=\omega^{2}$. Thus, we have the equality $\Gamma_{v\to x}=\Gamma_{x\to v}=\omega$ and $E_{x\to v}=E_{v\to x}=\omega^{2}$, as alluded to in the discussion following Equation (35).

## 4. Entropy-Based Causality Measures

As noted previously, most of information theoretical measures of causality are based on entropy, joint entropy, conditional entropy, or mutual information, etc. Specifically, for the two dependent stochastic variables $X_{1}$ and $X_{2}$ with the marginal PDFs $p(X_{1},t)$ and $p(X_{2},t)$ and joint PDF $p(X_{1},t_{1};X_{2},t_{2})$, entropy $S(X_{1})$, joint entropy $S(X_{1},X_{2})$, mutual entropy $S(X_{1}|X_{2})$, and mutual information $I(X_{1},X_{2})$ are defined by

(58) $$\begin{array}{ccc} S(X_{1}\left(t_{1}\right)) & = & -\int dX_{1}p\left(X_{1},t_{1}\right)\ln p(X_{1},t_{1}),\;\;\; \end{array}$$

(59) $$\begin{array}{ccc} S(X_{2}\left(t_{2}\right)) & = & -\int dX_{2}p\left(X_{2},t_{2}\right)\ln p(X_{2},t_{2}), \end{array}$$

(60) $$\begin{array}{ccc} S(X_{1}\left(t_{1}\right),X_{2}\left(t_{2}\right)) & = & -\int dX_{1}dX_{2}\;p\left(X_{1},t_{1};X_{2},t_{2}\right)\ln p(X_{1},t_{1};X_{2},t_{2}), \end{array}$$

(61) $$\begin{array}{ccc} S(X_{1}\left(t_{1}\right)|X_{2}\left(t_{2}\right)) & = & S\left(X_{1}\left(t_{1}\right),X_{2}\left(t_{2}\right)\right)-S\left(X_{2}\left(t_{2}\right)\right), \end{array}$$

(62) $$\begin{array}{ccc} I(X_{1}\left(t_{1}\right):X_{2}\left(t_{2}\right)) & = & S\left(X_{1}\left(t_{1}\right)\right)-S\left(X_{1}\left(t_{1}\right),X_{2}\left(t_{2}\right)\right)\\ & = & S\left(X_{1}\left(t_{1}\right)\right)+S\left(X_{2}\left(t_{2}\right)\right)-S\left(X_{1}\left(t_{1}\right),X_{2}\left(t_{2}\right)\right). \end{array}$$

For Gaussian processes, Equations (13)–(15) show that the entropy depends only on the variance/covariance, being independent of the mean value. This can be problematic as entropy fails to capture the effect of one variable on the mean value of another variable, for instance, caused by rare events associated with coherent structures, such as vortices, shear flows, etc. This is explicitly shown in Section 5.4 (see Figure 5) in regard to causality. Although not widely recognized, it is important to point out the limitation of entropy-based measures in measuring perturbations (in particular, caused by abrupt events) that do not affect entropy, as shown in Reference [32]. In addition, entropy has shortcomings, such as being non-invariant under coordinate transformations and insensitive to the local arrangement (shape) of p(x,t) for fixed *t*. Similar comments are applicable to other entropy-based measures. To demonstrate this point, in this section, we provide a detailed analysis of information flow based on conditional entropy [16,41].

### 4.1. $T_{v\to x}$

Information flow is based on predicting gain (or loss) of the future of subsystem 1 from the present state of subsystems 2 and defined as

(63) $$\begin{array}{ccc} T_{v\to x} & = & \lim_{t_{1}\to t^{+}}\partial_{t_{1}}I\left(x\left(t_{1}\right):v\left(t\right)\right)\\ & = & \frac{dS(x(t))}{dt}-\lim_{t_{1}\to t^{+}}\partial_{t_{1}}S\left(x\left(t_{1}\right)|v\left(t\right)\right)=\frac{dS(x(t))}{dt}-\lim_{t_{1}\to t^{+}}\partial_{t_{1}}S\left(x\left(t_{1}\right),v\left(t\right)\right), \end{array}$$

where Equation (62) is used. Here, the first term and the second term on the RHS represent the rate of change of the marginal entropy of x(t) and the rate of change of the conditional entropy of $x(t_{1})$ conditional on v(t) (i.e., frozen *v*). The difference between these two rates then quantifies the effect of the evolution of *v* on the entropy of *x*. Note that $T_{v\to x}$ can be both negative and positive; a negative $T_{v\to x}$ means that *v* acts to reduce the marginal entropy of *x* ($S_{1}$), as numerically observed in Reference [32].

Using Equation (13), we have $\partial_{t_{1}}S\left(x\left(t_{1}\right),v\left(t\right)\right)=\frac{\partial_{t_{1}}\left|\Sigma \left(t_{1},t\right)\right|}{\left|\Sigma (t_{1},t)\right|}$. Then, by using Equations (26)–(28) and (29), we obtain

(64) $$\begin{array}{ccc} \lim_{t_{1}\to t}\partial_{t_{1}}\left|\Sigma \left(t_{1},t\right)\right| & = & {\dot{\Sigma }}_{xx}\Sigma_{vv}-2{\dot{\Sigma }}_{xv}\Sigma_{xv}=2\Sigma_{xv}\Sigma_{vv}-2\Sigma_{vv}\Sigma_{xv}=0, \end{array}$$

and

(65) $$\begin{array}{ccc} T_{v\to x} & = & \frac{dS(x(t))}{dt}=\frac{1}{2}\frac{{\dot{\Sigma }}_{xx}}{\Sigma_{xx}}=\frac{\Sigma_{xv}}{\Sigma_{xx}}. \end{array}$$

As can be seen from Equation (65), $T_{v\to x}$ depends only on the variance, being independent of the mean value. Furthermore, $T_{v\to x}$ is proportional to the cross-correlation $\Sigma_{xv}$, becoming zero for $\Sigma_{xv}=0$ as in the case of equilibrium. (Note that Equation (65) is derived using a different method in Reference [32] for the Kramers equation.)

### 4.2. $T_{x\to v}$

Similarly, information flow is based on predicting gain (or loss) of the future of subsystem 2 from the present state of subsystems 1 and defined as

(66) $$\begin{array}{ccc} T_{x\to v} & = & \lim_{t_{1}\to t^{+}}\partial_{t_{1}}I\left(x\left(t\right):v\left(t_{1}\right)\right)\\ & = & \frac{dS(v(t))}{dt}-\lim_{t_{1}\to t^{+}}\partial_{t_{1}}S\left(x\left(t\right)|v\left(t_{1}\right)\right)=\frac{dS(v(t))}{dt}-\lim_{t_{1}\to t^{+}}\partial_{t_{1}}S\left(x\left(t\right),v\left(t_{1}\right)\right), \end{array}$$

where Equation (62) is used. Here, the first term and the second term on the RHS represent the rate of change of the marginal entropy of v(t) and the rate of change of the conditional entropy of $v(t_{1})$ conditional on x(t) (i.e., frozen *x*). The difference between these two rates then quantifies the effect of the evolution of *x* on the entropy of *v*. Note again that $T_{x\to v}$ can be both negative and positive; a negative $T_{x\to v}$ means that *x* acts to reduce the marginal entropy of *v* ($S_{2}$), as numerically observed in Reference [32].

For $\partial_{t_{1}}S\left(x\left(t\right),v\left(t_{1}\right)\right]=\frac{\partial_{t_{1}}\left|\Sigma \left(t,t_{1}\right)\right|}{\left|\Sigma (t,t_{1})\right|}$, we use Equations (44)–(48) and (50) to obtain

(67) $$\begin{array}{ccc} \lim_{t_{1}\to t}\partial_{t_{1}}\left|\Sigma \left(t,t_{1}\right)\right| & = & {\Sigma^{\prime }}_{vv}\left(t\right)\Sigma_{xx}\left(t\right)-2{\Sigma^{\prime }}_{xv}\left(t\right)\Sigma_{xv}\\ & = & \gamma \left(-2\Sigma_{xx}\Sigma_{vv}+2\Sigma_{xv}^{2}\right)+2D\Sigma_{xx}=-2\gamma \left|\Sigma \right|+2D\Sigma_{xx}. \end{array}$$

Thus,

(68) $$\begin{array}{ccc} T_{x\to v} & = & \frac{dS(v(t))}{dt}-\lim_{t_{1}\to t^{+}}\partial_{t_{1}}S\left(X_{1}\left(t\right),X_{2}\left(t_{1}\right)\right)\\ & = & \frac{1}{2}\left[\frac{{\Sigma^{\prime }}_{vv}}{\Sigma_{vv}}-\frac{-2\gamma |\Sigma |+2D\Sigma_{xx}}{\left|\Sigma \right|}\right]=-\omega^{2}\frac{\Sigma_{xv}}{\Sigma_{vv}}-D\frac{{\left(\Sigma_{xv}\right)}^{2}}{\Sigma_{vv}\left|\Sigma \right|}. \end{array}$$

Again, Equation (68) is derived using a different method in Reference [32]. As in the case of $T_{v\to x}$ in Equation (65), $T_{x\to v}$ depends only on the variance, being independent of the mean value while being proportional to the cross-correlation $\Sigma_{xv}$, becoming zero for $\Sigma_{xv}=0$ as in the case of equilibrium.

## 5. Comparisons between Causal Information Rate and Information Flow

In this section, we compare the causal information rate in Equations (33) and (54) with the information flow in Equations (65) and (68) for the Kramers equation by focusing on several interesting cases. We start by noting that if γ≠0, ω≠0, and D≠0, *x* and *v* evolve to equilibrium where the covariance matrix takes the values:

(69) $$\Sigma_{xx}=\frac{D}{\gamma \omega^{2}},\;\;\;\Sigma_{vv}=\frac{D}{\gamma },\;\;\;\Sigma_{xv}=0.$$

**Figure 1:**
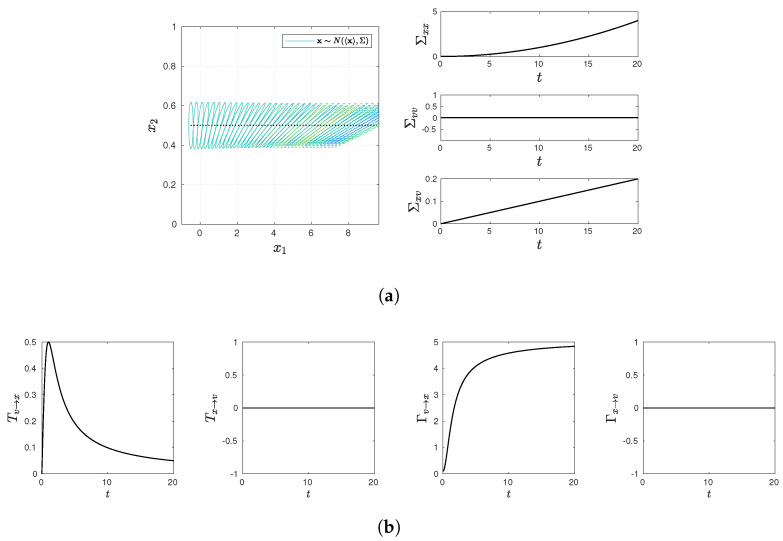
Snapshots of PDFs, $\Sigma_{xx}\left(t\right)$, $\Sigma_{vv}\left(t\right)$, and $\Sigma_{xv}\left(t\right)$ in panel (**a**) (PDF snapshots are shown for one-standard deviation, using different colors for different times); $T_{x\to v}$, $T_{v\to x}$, $\Gamma_{x\to v}$, $\Gamma_{v\to x}$ in panel (**b**). Parameter values are D=0, γ=0, and ω=0.

**Figure 2:**
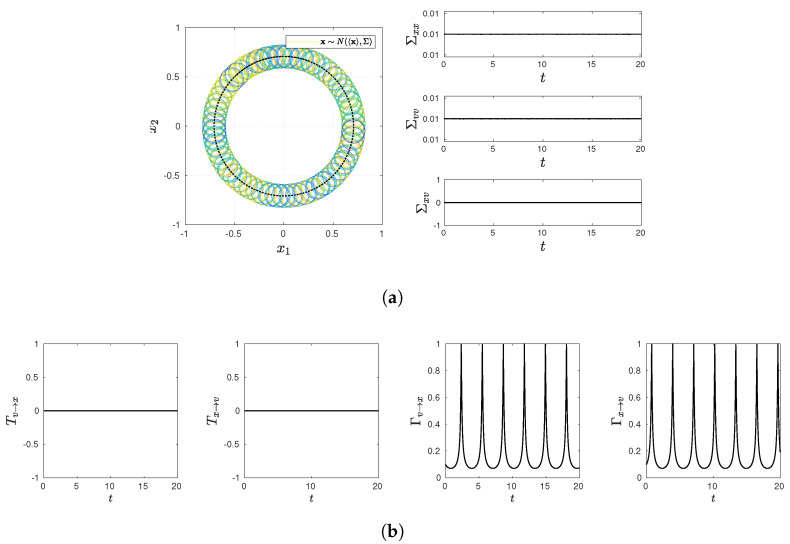
Snapshots of PDFs, $\Sigma_{xx}\left(t\right)$, $\Sigma_{vv}\left(t\right)$, and $\Sigma_{xv}\left(t\right)$ in panel (**a**) (PDF snapshots are shown for one-standard deviation, using different colors for different times); $T_{x\to v}$, $T_{v\to x}$, $\Gamma_{x\to v}$, $\Gamma_{v\to x}$ in panel (**b**). Parameter values are D=0, γ=0, and ω=1.

**Figure 3:**
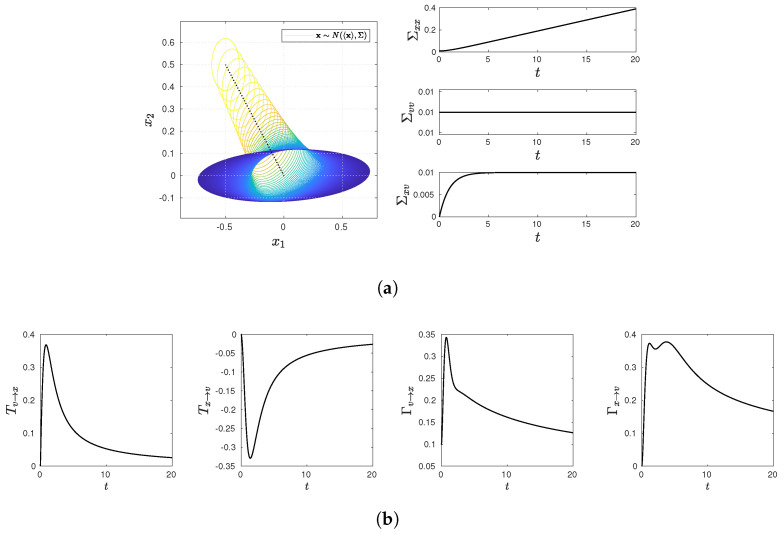
Snapshots of PDFs, $\Sigma_{xx}\left(t\right)$, $\Sigma_{vv}\left(t\right)$, and $\Sigma_{xv}\left(t\right)$ in panel (**a**) (PDF snapshots are shown for one-standard deviation, using different colors for different times); $T_{x\to v}$, $T_{v\to x}$, $\Gamma_{x\to v}$, $\Gamma_{v\to x}$ in panel (**b**). Parameter values are D=0.01, γ=1, and ω=0.

**Figure 4:**
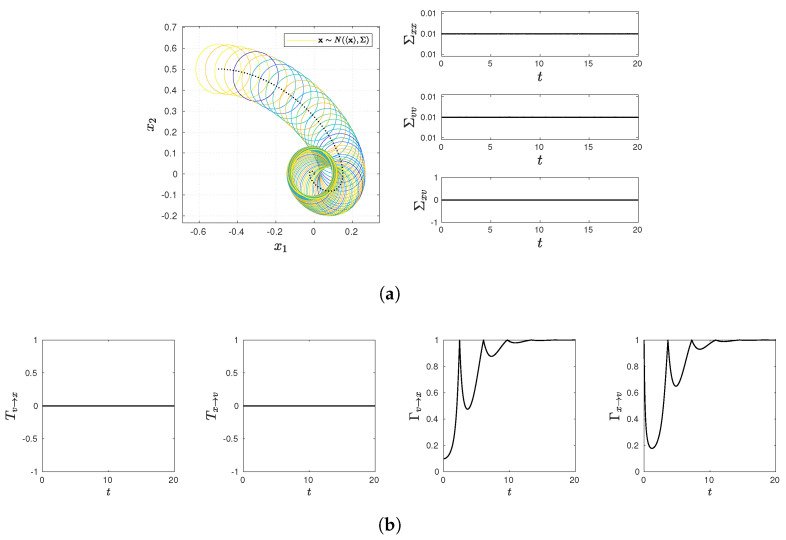
Snapshots of PDFs, $\Sigma_{xx}\left(t\right)$, $\Sigma_{vv}\left(t\right)$, and $\Sigma_{xv}\left(t\right)$ in panel (**a**) (PDF snapshots are shown for one-standard deviation, using different colors for different times); $T_{x\to v}$, $T_{v\to x}$, $\Gamma_{x\to v}$, $\Gamma_{v\to x}$ in panel (**b**). Parameter values are D=0.01, γ=1, ω=1.

**Figure 5:**
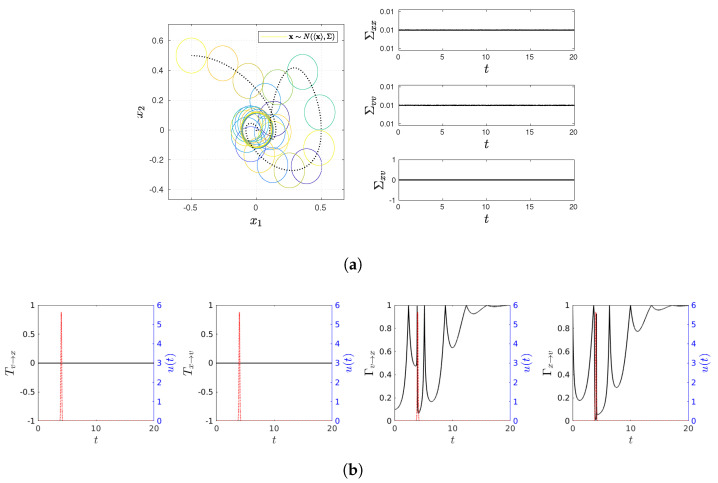
Snapshots of PDFs, $\Sigma_{xx}\left(t\right)$, $\Sigma_{vv}\left(t\right)$, and $\Sigma_{xv}\left(t\right)$ in panel (**a**) (PDF snapshots are shown for one-standard deviation, using different colors for different times); $T_{x\to v}$, $T_{v\to x}$, $\Gamma_{x\to v}$, $\Gamma_{v\to x}$ in panel (**b**). Parameter values are D=0.01, γ=1, ω=1, with an impulse u(t)≠0 with c=0.1 and $t_{0}=4$.

We use the same initial conditions

(70) $$\langle x\left(0\right)\rangle =-0.5,\;\langle v\left(0\right)\rangle =0.5,\;\Sigma_{xx}\left(0\right)=\Sigma_{vv}\left(0\right)=0.01,\;\Sigma_{xv}\left(0\right)=0,$$

and present various statistical quantifies in Figure 1, Figure 2, Figure 3, Figure 4 and Figure 5, including snapshots of PDFs, $\Sigma_{xx}\left(t\right)$, $\Sigma_{vv}\left(t\right)$, and $\Sigma_{xv}\left(t\right)$ in panel (a); $T_{x\to v}$, $T_{v\to x}$, $\Gamma_{x\to v}$, $\Gamma_{v\to x}$ in panel (b). Note that PDF snapshots are shown for one-standard deviation, using different colors for different times.

### 5.1. No Stochastic Noise D=0

It is useful to look at the deterministic case without the stochastic noise ξ=0 in Equations (7) and (8), where a time-dependent PDF evolves due to non-zero initial conditions. Specifically, the two cases where γ=ω=0 and γ=0 and ω=1 are considered in Figure 1 and Figure 2, respectively.

To gain a key insight into the meaning of our causal information rate, we start with the simplest case in Figure 1, where γ=0=ω, with *v* being fixed to its initial value v(0) so that x(t)=x(0)+v(0)t. The snapshots of the PDF and the covariance matrix in Figure 1a show that the PDF center (peak) undergoes a drift according to 〈x(t)〉=〈x(0)〉+〈v(0)〉t, while the PDF broadens with time in the *x*-direction since δx(t)=δx(0)+tδv(0). As a result, $\Sigma_{xv}$ increases linearly with time. $\Sigma_{xv}\neq 0$ causes a rapid initial increase in information flow $T_{v\to x}\neq 0$ in Figure 1b. However, as t→∞, $T_{v\to x}\to 0$ since $\Sigma_{xx}$ increases faster than $\Sigma_{xv}$ in time, leading to $T_{v\to x}=\frac{\Sigma_{xv}}{\Sigma_{xx}}\to 0$ (see Equation (65)). Thus, $T_{v\to x}$ fails to reflect the feedback from *v* to *x* in the long time limit. In contrast, $\Gamma_{v\to x}$ monotonically increases with time, approaching a constant value ($\Gamma_{v\to x}\to 5$) as t→∞. On the other hand, $\Gamma_{x\to v}=T_{x\to v}=0$ at all times, reflecting the lack of the coupling from *x* to *v* at all times, consistent with our expectation. That is, the lack of the feedback from *x* on *v* is reflected in both $\Gamma_{x\to v}=T_{x\to v}=0$, while the one-way coupling of *v* to *x* is captured only by $\Gamma_{v\to x}\neq 0$ at any time.

To include the feedback of *x* on *v*, we now consider the case γ=0 and ω=1 in Figure 2. Non-zero value of ω (=1)—only the difference from Figure 1—now establishes the two-way (mutual) communications between *x* and *v*, leading to the harmonic motion (see Equations (7) and (8)). Figure 2a shows how the PDF center drifts according to this harmonic motion, while the cross-correlation $\Sigma_{xv}\left(t\right)=0$ at any time. The latter leads to the information flow $T_{x\to v}=T_{v\to x}=0$ shown in Figure 2b. In contrast, $\Gamma_{x\to v}$ and $\Gamma_{v\to x}$ in Figure 2b exhibit oscillations with a 90 degree phase-shift between the two due to the harmonic motion, capturing the two-way feedback between *x* and *v*. To highlight an exact symmetry between $\Gamma_{x\to v}$ and $\Gamma_{v\to x}$, we can consider a global, path-dependent measure of causality by integrating $\Gamma_{x\to v}$ and $\Gamma_{v\to x}$ over the same integer multiples of the period (2π/ω), which would give the same value. These results reveal that our causal information rate captures the dependence between *x* and *v* even in the absence of their cross-correlation. (We recall that zero cross-correlation does not imply independence.)

### 5.2. Equilibrium: v

We now consider the Ornstein-Uhlenbeck (O-U) process of *v* by choosing γ=1, ω=0 and D≠0 in Figure 3. In this case, *v* approaches asymptotically its equilibrium distribution where $\Sigma_{vv}=\frac{D}{\gamma }$, while p(x,t) evolves in time. Specifically, we choose $D=D_{xx}\left(0\right)=D_{vv}\left(0\right)=0.01$ (see also Equation (70)). Figure 3a shows that the PDF broadens in the *x*-direction ($\Sigma_{xx}\propto t$), while keeping its original width in the *v*-direction. On the other hand, due to the non-zero D≠0, the cross-correlation $\Sigma_{xv}\left(t\right)>0$ is seen to grow in time, approaching a constant value 0.01 as t→∞. As in the case of Figure 2, $T_{x\to v}$ and $T_{v\to x}$ take finite values due to non-zero $\Sigma_{xv}\neq 0$ but become zero asymptotically as t→∞ (due to $\Sigma_{xx}\propto t$), as shown in Figure 3b. On the other hand, the behavior of $\Gamma_{x\to v}$ and $\Gamma_{v\to x}$ is quite similar.

### 5.3. Equilibrium: x and v

To ensure a quick evolution to the equilibrium distribution in time, we choose D=0.01, ω=1, and γ=1 so that the initial and final (equilibrium) PDFs have the same variance in Figure 4. Figure 4a shows that the PDF center undergoes a damped oscillation without changing its shape since $\Sigma_{xx}\left(t\right)=\Sigma_{vv}\left(t\right)=0.01$ and $\Sigma_{xv}\left(t\right)=0$ at all times. $\Sigma_{xv}\left(t\right)=0$ leads to $T_{x\to v}=T_{v\to x}=0$ in Figure 4b, as in the case of Figure 2b. In contrast, the drift of the PDF center leads to non-zero values of $\Gamma_{v\to x}$ and $\Gamma_{x\to v}$, which asymptotically approach 1 (see Equations (57)). The latter reveals that the equilibrium is maintained through the two-way communications between *x* and *v*.

### 5.4. An Abrupt Change Introduced by an Impulse

In Reference [32], we showed that an abrupt event (modeled by an impulse function, with a peak at a certain time) caused a sudden increase in $E=\Gamma^{2}$ in all cases, while it caused a sudden increase in the magnitude of the information flow $|T_{i\to j}|$ (i≠j) only when the perturbation affects entropy (variance). Furthermore, it was shown that the peak of $|T_{i\to j}|$ (i≠j) followed (not preceded) the actual impulse peak, while the peak of $E=\Gamma^{2}$ tended to precede the impulse peak. This means that, by measuring the temporal change in E, especially, when its peak appears, we can forecast the onset of an abrupt event (whose peak appears later than E-peak in time). We now look at the effect of a sudden impulse on the causal information rate.

To this end, we introduce a sudden perturbation to the Kramers equation by adding an impulse function u(t) as a time-dependent additive force to Equation (8) as follows:

(71) $$\begin{array}{ccc} \frac{dv}{dt} & = & -\gamma v-\omega^{2}x+u\left(t\right)+\xi ,\\ u(t) & = & \frac{1}{c\sqrt{\pi }}e^{-{\left(\frac{t-t_{0}}{c}\right)}^{2}}. \end{array}$$

We use the analytical expressions for the mean values and covariance in Reference [32] and choose the parameter values c=0.1 and $t_{0}=4$ in Equation (71) and D=ω=γ=1 (the same as in Figure 4). The results are shown in Figure 5, where the impulse function u(t) localized around t=4 is shown in red dotted line, using the right *y* axis, in the bottom panels in Figure 5b.

Figure 5a shows that u(t) causes a sudden drift of the PDF center with no change in variance. Therefore, $T_{x\to v}\left(t\right)=T_{v\to x}\left(t\right)=0$ in Figure 5b, with no influence of u(t). In sharp contrast, $\Gamma_{x\to v}$ and $\Gamma_{v\to x}$ exhibit abrupt change around time t=4. Furthermore, the peak in $\Gamma_{x\to v}$ (or $\Gamma_{v\to x}$) tends to proceed the impulse peak (in red dotted line). We observe similar results when an impulse is applied to the covariance matrix (results not shown). These results, thus, suggest that our causality information rate is sensitive to the perturbation (in both mean and variance) and predicts the onset of a sudden event very well, especially in comparison with the information flow. We emphasize that the information flow (and other entropy-based methods) cannot detect the onset of a sudden event that does not affect entropy (e.g., Reference [32] for different examples).

## 6. Conclusions

Information geometry in general concerns the distinguishability between two PDFs (e.g., constructed from data) and is sensitive to the local dynamics (e.g., Reference [27]), depending on a local arrangement (the shape) of the PDFs. This is different from entropy, which is a global measure of a PDF, being insensitive to such a local arrangement. When a PDF is a continuous function of time, the information rate and information length are helpful in understanding far-from-equilibrium phenomena in terms of the number of distinguishable statistical states that a system evolves through in time. Being very sensitive to evolving dynamics, it enables us to compare different far-from-equilibrium processes using the same dimensionless distance, as well as quantifying the relation (correlation, self-regulation, etc.) among variables (e.g., References [27,28,29,30]).

In this paper, by extending our previous work [20,21,22,23,24,25,26,27,28,29,30,31], we introduced the causal information rate as a general information-geometric method that can elucidate causality relations in stochastic processes involving temporal variabilities and strong fluctuations. The key idea was to quantify the effect of one variable on the information rate of the other variable. The cross-correlation between the variables was shown to play a key role in the information flow, zero cross-correlation leading to zero information flow. In comparison, the causal information rate can take a non-zero value in the absence of cross-correlation. Since zero cross-correlation (measuring only the linear dependence) does not imply independence in general, this means that the causal information rate captures the (directional) dependence between two variables even when they are uncorrelated with each other.

Furthermore, the causal information rate captures the temporal change in both covariance matrix and mean value. In comparison, the information flow depends only on the temporal change in the covariance matrix. Thus, the causal information rate is a sensitive method for predicting an abrupt event and quantifying causal relations. These properties are welcome for predicting rare, large-amplitude events. Application has been made to the Kramers equation to highlight these points. Although the analysis in this paper is limited to the Gaussian variables that are entirely characterized by the mean and variance, similar results are likely to hold for non-Gaussian variables because the information rate captures the temporal changes of a PDF itself, while entropy-based measures (e.g., information flow) depend only on variance.

Given that causality (directional dependence) plays a crucial role in science and engineering (e.g., References [43,44]), our method could be useful in a wide range of problems. In particular, it could be utilized to elucidate causal relations among different players in nonlinear dynamical systems, fluid/plasma dynamics, laboratory plasmas, astrophysical systems, environmental science, finance, etc. For instance, in fluid/plasmas turbulence, it could help resolving the controversy over causality in the low-to-high (L-H) confinement transition [29,30,45,46], as well as contributing to identifying a causal relationship among different players responsible for the onset of sudden abrupt events (e.g., fusion plasmas eruption) (e.g., References [47,48]), with a better chance of control. It could also elucidate causal relationships among different physiological signals, how different parts of a human body (e.g., brain-heart-connection) are self-regulated to maintain homeostasis (the optimal living condition for survival), and how this homeostasis degrades with the onset of diseases.

Finally, it will be interesting to investigate the effects of coarse-graining in future works. In Reference [49], for the information geometry given by the Fisher metric, relevant directions were shown to be exactly maintained under coarse-graining, while irrelevant directions contract. The analysis for more than two variables will also be addressed in future work.

## Appendix A. Solutions to the Kramers Equations

The general solution to the Kramers equation in Equations (7)–(9) is

(A1) $$\begin{array}{ccc} x(t) & = & x_{h}\left(t\right)+\frac{1}{\Delta }\int_{0}^{t}dt_{1}\;\left[e^{\alpha_{+}t_{1}}-e^{\alpha_{-}t_{1}}\right]\xi \left(t_{1}\right), \end{array}$$

(A2) $$\begin{array}{ccc} v(t) & = & v_{h}\left(t\right)+\frac{1}{\Delta }\int_{0}^{t}dt_{1}\;\left[\alpha_{+}e^{\alpha_{+}t_{1}}-\alpha_{-}e^{\alpha_{-}t}\right]\xi \left(t_{1}\right), \end{array}$$

where $x_{h}\left(t\right)$ and $v_{h}\left(t\right)$ are the homogeneous solution and

(A3) $$\alpha_{\pm }=\frac{1}{2}\left[-\gamma \pm \sqrt{\gamma^{2}-4\omega^{2}}\right]\equiv \frac{1}{2}\left[-\gamma \pm \Delta \right],$$

where $\Delta =\gamma^{2}-4\omega^{2}$. For the initial condition $x\left(t=0\right)=x_{0}$ and $v\left(t=0\right)=v_{0}$, they are

(A4) $$\begin{array}{ccc} x_{h}\left(t\right) & = & \frac{1}{\Delta }\left[\left(-\alpha_{-}e^{\alpha_{+}t}+\alpha_{+}e^{\alpha_{-}t}\right)x\left(0\right)+\left(e^{\alpha_{+}t}-e^{\alpha_{-}t}\right)v\left(0\right)\right], \end{array}$$

(A5) $$\begin{array}{ccc} v_{h}\left(t\right) & = & \frac{1}{\Delta }\left[\alpha_{+}\alpha_{-}\left(-e^{\alpha_{+}t}+e^{\alpha_{-}t}\right)x\left(0\right)+\left(\alpha_{+}e^{\alpha_{+}t}-\alpha_{-}e^{\alpha_{-}t}\right)v\left(0\right)\right]. \end{array}$$

By taking the averages of Equations (A1) and (A2) with the help of Equations (A4) and (A5), we obtain the evolution of the mean values as

(A6) $$\begin{array}{ccc} \langle x(t)\rangle & = & \frac{1}{\Delta }\left[\left(-\alpha_{-}e^{\alpha_{+}t}+\alpha_{+}e^{\alpha_{-}t}\right)\langle x\left(0\right)\rangle +\left(e^{\alpha_{+}t}-e^{\alpha_{-}t}\right)\langle v\left(0\right)\rangle \right], \end{array}$$

(A7) $$\begin{array}{ccc} \langle v(t)\rangle & = & \frac{1}{\Delta }\left[\alpha_{+}\alpha_{-}\left(-e^{\alpha_{+}t}+e^{\alpha_{-}t}\right)\langle x\left(0\right)\rangle +\left(\alpha_{+}e^{\alpha_{+}t}-\alpha_{-}e^{\alpha_{-}t}\right)\langle v\left(0\right)\rangle \right]. \end{array}$$

The elements of the equal-time covariance matrix Σ(t) are obtained by subtracting Equations (A6) and (A7) from Equations (A1) and (A2), respectively, and then by multiplying and taking averages using Equation (9):

(A8) $$\begin{array}{ccc} \Sigma_{xx} & = & \langle {\left(\delta x\left(t\right)\right)}^{2}\rangle =\langle {\left(\delta x_{h}\left(t\right)\right)}^{2}\rangle +\frac{2D}{\Delta^{2}}\int_{0}^{t}dt_{1}\;{\left[e^{\alpha_{+}t_{1}}-e^{\alpha_{-}t_{1}}\right]}^{2}\\ & = & \langle {\left(\delta x_{h}\left(t\right)\right)}^{2}\rangle +\frac{D}{\Delta^{2}}\left[\frac{1}{\alpha_{+}}\left[e^{2\alpha_{+}t}-1\right]+\frac{1}{\alpha_{-}}\left[e^{2\alpha_{-}t}-1\right]-\frac{4}{\alpha_{+}+\alpha_{-}}\left[e^{(\alpha_{+}+\alpha_{-})t}-1\right]\right], \end{array}$$

(A9) $$\begin{array}{ccc} \Sigma_{vv} & = & \langle {\left(\delta x\left(t\right)\right)}^{2}\rangle =\langle {\left(\delta v_{h}\left(t\right)\right)}^{2}\rangle +\frac{2D}{\Delta^{2}}\int_{0}^{t}dt_{1}\;{\left[\alpha_{+}e^{\alpha_{+}t_{1}}-\alpha_{-}e^{\alpha_{-}t_{1}}\right]}^{2}\\ & = & \langle {\left(\delta v_{h}\left(t\right)\right)}^{2}\rangle +\frac{D}{\Delta^{2}}\left[\alpha_{+}\left[e^{2\alpha_{+}t}-1\right]+\alpha_{-}\left[e^{2\alpha_{-}t}-1\right]-\frac{4\alpha_{+}\alpha_{-}}{\alpha_{+}+\alpha_{-}}\left[e^{(\alpha_{+}+\alpha_{-})t}-1\right]\right], \end{array}$$

(A10) $$\begin{array}{ccc} \Sigma_{xv} & = & \langle \delta x\left(t\right)\delta v\left(t\right)\rangle =\langle \delta x_{h}\left(t\right)\delta v_{h}\left(t\right)\rangle +\frac{2D}{\Delta^{2}}\int_{0}^{t}dt_{1}\;\left[e^{\alpha_{+}t_{1}}-e^{\alpha_{-}t_{1}}\right]\left[\alpha_{+}e^{\alpha_{+}t_{1}}-\alpha_{-}e^{\alpha_{-}t_{1}}\right]\\ & & =\langle \delta x_{h}\left(t\right)\delta v_{h}\left(t\right)\rangle +\frac{4De^{-\gamma t}}{\Delta^{2}}\sinh^{2}\left(\frac{\Delta t}{2}\right). \end{array}$$

Here,

(A11) $$\begin{array}{ccc} \langle {\left(\delta x_{h}\left(t\right)\right)}^{2}\rangle & = & \frac{1}{\Delta^{2}}[{\left(-\alpha_{-}e^{\alpha_{+}t}+\alpha_{+}e^{\alpha_{-}t}\right)}^{2}\Sigma_{xx}\left(0\right)+{\left(e^{\alpha_{+}t}-e^{\alpha_{-}t}\right)}^{2}\Sigma_{vv}\left(0\right)\\ & & +2\left(e^{\alpha_{+}t}-e^{\alpha_{-}t}\right)\left(-\alpha_{-}e^{\alpha_{+}t}+\alpha_{+}e^{\alpha_{-}t}\right)\Sigma_{xv}\left(0\right)], \end{array}$$

(A12) $$\begin{array}{ccc} \langle {\left(\delta v_{h}\left(t\right)\right)}^{2}\rangle & = & \frac{1}{\Delta^{2}}[\alpha_{+}^{2}\alpha_{-}^{2}{\left(-e^{\alpha_{+}t}+e^{\alpha_{-}t}\right)}^{2}\Sigma_{xx}\left(0\right)+{\left(\alpha_{+}e^{\alpha_{+}t}-\alpha_{-}e^{\alpha_{-}t}\right)}^{2}\Sigma_{vv}\left(0\right)\\ & & +2\alpha_{+}\alpha_{-}\left(-e^{\alpha_{+}t}+e^{\alpha_{-}t}\right)\left(\alpha_{+}e^{\alpha_{+}t}-\alpha_{-}e^{\alpha_{-}t}\right)\Sigma_{xv}\left(0\right)], \end{array}$$

(A13) $$\begin{array}{ccc} \langle \delta x_{h}\left(t\right)\delta v_{h}\left(t\right)\rangle & = & \frac{1}{\Delta^{2}}[\alpha_{+}\alpha_{-}\left(-\alpha_{-}e^{\alpha_{+}t}+\alpha_{+}e^{\alpha_{-}t}\right)\left(-e^{\alpha_{+}t}+e^{\alpha_{-}t}\right)\Sigma_{xx}\left(0\right)\\ & & +\left[-\alpha_{+}\alpha_{-}{\left(e^{\alpha_{+}t}-e^{\alpha_{-}t}\right)}^{2}+\left(\alpha_{+}e^{\alpha_{+}t}-\alpha_{-}e^{\alpha_{-}t}\right)\left(-\alpha_{-}e^{\alpha_{+}t}+\alpha_{+}e^{\alpha_{-}t}\right)\Sigma_{xv}\left(0\right)\right]\\ & & +\left(e^{\alpha_{+}t}-e^{\alpha_{-}t}\right)\left(\alpha_{+}e^{\alpha_{+}t}-\alpha_{-}e^{\alpha_{-}t}\right)\Sigma_{vv}\left(0\right)], \end{array}$$

where $\Sigma_{xx}\left(0\right)=\langle {\left(\delta x\left(0\right)\right)}^{2}\rangle$, $\Sigma_{vv}\left(0\right)=\langle {\left(\delta v\left(0\right)\right)}^{2}\rangle$, and $\Sigma_{xv}\left(0\right)=\langle \delta x\left(0\right)\delta v\left(0\right)\rangle$.

## Appendix B. Derivation of Equation (25)

We let $\phi =\frac{1}{2}\Sigma_{ij}^{-1}\left(t_{1},t\right)\left(X_{i}-\langle X_{i}\rangle \right)\left(X_{j}-\langle X_{j}\rangle \right)$ so that Equation (21) becomes

(A14) $$\begin{array}{ccc} p(x,t_{1};v,t) & = & \frac{1}{2\pi \sqrt{\left|\Sigma (t_{1},t)\right|}}\exp\left(-\phi \right). \end{array}$$

Then, we have

(A15) $$\begin{array}{ccc} \frac{\partial_{t_{1}}p}{p} & = & -\frac{\dot{\left|\Sigma \right|}}{2|\Sigma |}-\partial_{t_{1}}\phi , \end{array}$$

(A16) $$\begin{array}{ccc} {\left(\frac{\partial_{t_{1}}p}{p}\right)}^{2} & = & \frac{{\dot{\left|\Sigma \right|}}^{2}}{{4|\Sigma |}^{2}}+\partial_{t_{1}}\phi \frac{\dot{\left|\Sigma \right|}}{\left|\Sigma \right|}+{\left(\partial_{t_{1}}\phi \right)}^{2}, \end{array}$$

(A17) $$\begin{array}{ccc} \int dxdv\;p{\left(\frac{\partial_{t_{1}}p}{p}\right)}^{2} & = & \frac{{\dot{\left|\Sigma \right|}}^{2}}{{4|\Sigma |}^{2}}+\langle \partial_{t_{1}}\phi \rangle \frac{\dot{\left|\Sigma \right|}}{\left|\Sigma \right|}+\langle {\left(\partial_{t_{1}}\phi \right)}^{2}\rangle , \end{array}$$

where $\dot{\Sigma }=\partial_{t_{1}}\Sigma$. Using $\partial_{t_{1}t_{1}}e^{-\phi }=-\left(\partial_{t_{1}t_{1}}\phi \right)e^{-\phi }+{\left(\partial_{t_{1}}\phi \right)}^{2}e^{-\phi }$ and $\int dxdve^{-\phi }=2\pi \sqrt{\left|\Sigma \right|}$, we have

(A18) $$\begin{array}{ccc} \langle \partial_{t_{1}}\phi \rangle & = & -\frac{1}{2\pi \sqrt{\left|\Sigma \right|}}\partial_{t_{1}}\int dxdve^{-\phi }=-\frac{\dot{\left|\Sigma \right|}}{2|\Sigma |}, \end{array}$$

(A19) $$\begin{array}{ccc} \langle {\left(\partial_{t_{1}}\phi \right)}^{2}\rangle & = & -\frac{1}{4}\frac{{\dot{\left|\Sigma \right|}}^{2}}{{\left|\Sigma \right|}^{2}}+\frac{1}{2}\frac{\ddot{\left|\Sigma \right|}}{\left|\Sigma \right|}+\langle \partial_{t_{1}t_{1}}\phi \rangle . \end{array}$$

Thus, by using Equations (A18) and (A19) in Equation (A17), we have

(A20) $$\begin{array}{ccc} E_{x}^{*}\left(t\right) & = & \lim_{t_{1}\to t^{+}}\left[\frac{1}{2}\partial_{t}\left(\frac{\dot{\left|\Sigma \right|}}{\left|\Sigma \right|}\right)+\langle \partial_{t_{1}t_{1}}\phi \rangle \right]=\lim_{t_{1}\to t^{+}}\left[\frac{1}{2}\mathrm{Tr}\left[\partial_{t_{1}}\left(\Sigma^{-1}\dot{\Sigma }\right)\right]+\langle \partial_{t_{1}t_{1}}\phi \rangle \right], \end{array}$$

where we used $\dot{\left|\Sigma \right|}=\left|\Sigma \right|\mathrm{Tr}\left(\Sigma^{-1}\dot{\Sigma }\right)$. To calculate $\langle \partial_{tt}\phi \rangle$, we use

(A21) $$\begin{array}{ccc} \partial_{t_{1}}\phi & = & \partial_{t_{1}}\left(\delta X_{i}\right)\Sigma_{ij}^{-1}\delta X_{j}+\frac{1}{2}\delta X_{i}\left(\partial_{t_{1}}\Sigma_{ij}^{-1}\right)\delta X_{j}, \end{array}$$

(A22) $$\begin{array}{ccc} \partial_{t_{1}t_{1}}\phi & = & \partial_{t_{1}t_{1}}\left(\delta X_{i}\right)\Sigma_{ij}^{-1}\delta X_{j}+\frac{1}{2}\delta X_{i}\left(\partial_{t_{1}t_{1}}\Sigma_{ij}^{-1}\right)\delta X_{j}+\partial_{t}\left(\delta X_{i}\right)\Sigma_{ij}^{-1}\partial_{t_{1}}\left(\delta X_{j}\right)\\ & & +2\partial_{t_{1}}\left(\delta X_{i}\right)\partial_{t_{1}}\left(\Sigma_{ij}^{-1}\right)\delta X_{j}, \end{array}$$

where i,j=1,2 and the symmetry $\Sigma_{ij}=\Sigma_{ji}$ is used. Since $\partial_{t}\left(\delta X_{i}\right)=-\langle \partial_{t}X_{i}\rangle$, etc., the average of Equation (A22) is simplified as

(A23) $$\begin{array}{ccc} \langle \partial_{t_{1}t_{1}}\phi \rangle & = & \partial_{t_{1}}\langle X_{i}\rangle \Sigma_{ij}^{-1}\partial_{t_{1}}\langle X_{j}\rangle +\frac{1}{2}\langle \delta X_{i}\left(\partial_{t_{1}t_{1}}\Sigma_{ij}^{-1}\right)\delta X_{j}\rangle \\ & = & \partial_{t}\langle X_{i}\rangle \Sigma_{ij}^{-1}\partial_{t}\langle X_{j}\rangle +\frac{1}{2}\mathrm{Tr}[\left(\partial_{t_{1}t_{1}}\Sigma^{-1})\Sigma \right], \end{array}$$

where $\langle \delta X_{i}G_{ij}\delta X_{j}\rangle =\mathrm{Tr}\left[G\Sigma \right]$ is used. Then, by using $\partial_{t_{1}}\Sigma^{-1}=-\Sigma^{-1}\dot{\Sigma }\Sigma^{-1}$ twice in Equation (A23), and putting the results in Equation (A20), we obtain Equation (25) in the text.

The derivation above is general. For the equal-time joint PDF, a similar analysis, or simply putting $t_{1}=t$ in Equation (25) gives us Equation (16). Furthermore, by taking $X_{i}$ and Σ to be a scalar as $X_{i}=x\delta_{i1}$ and $\Sigma =\Sigma_{xx}$ in Equation (16), we obtain Equation (17). Similarly, taking $X_{i}=v\delta_{i2}$ and $\Sigma =\Sigma_{vv}$ in Equation (16) gives us Equation (18).

## Appendix C. Comment on Equation (47)

In Equation (47), δv(t) consists of the homogeneous $\delta v_{h}\left(t\right)$ and inhomogeneous $\delta v_{I}\left(t\right)$ due to initial conditions and ξ, respectively, evolving according to

(A24) $$\begin{array}{ccc} \partial_{t}\delta v_{h}\left(t\right) & = & -\gamma \delta v_{h}\left(t\right)-\omega^{2}\delta v_{h}\left(t\right), \end{array}$$

(A25) $$\begin{array}{ccc} \partial_{t}\delta v_{I}\left(t\right) & = & -\gamma \delta v_{I}\left(t\right)-\omega^{2}\delta v_{I}\left(t\right)+\xi . \end{array}$$

Thus, by using Equations (A24) and (A25), we calculate ${\Sigma^{\prime }}_{vv}$ in Equation (47) as follows:

(A26) $$\begin{array}{ccc} {\Sigma^{\prime }}_{vv}\left(t\right) & = & \langle \left(\partial_{t}\delta v\left(t\right)\right)\delta v\left(t\right)\rangle =\langle \partial_{t}\left[\delta v_{h}\left(t\right)+\delta v_{I}\left(t\right)\right]\left(\delta v_{h}\left(t\right)+\delta v_{I}\left(t\right)\right)\rangle \\ & = & \langle \partial_{t}\left(\delta v_{h}\left(t\right)\right)\left(\delta v_{h}\left(t\right)\right))\rangle +\langle \partial_{t}\left(\delta v_{I}\left(t\right)\right)\left(\delta v_{I}\left(t\right)\right)\rangle \\ & = & -2\gamma \Sigma_{vv,h}-2\omega^{2}\Sigma_{xv,h}-2\gamma \Sigma_{vv,I}-2\omega^{2}\Sigma_{xv,I}+D\\ & = & -2\gamma \Sigma_{vv}-2\omega^{2}\Sigma_{xv}+D. \end{array}$$

Here, we used $\langle v_{h}\left(t\right)v_{I}\left(t\right)\rangle =0$; $\Sigma_{vv,h}=\langle \left(\delta v_{h}\left(t\right)\right)\left(\delta v_{h}\left(t\right)\right)\rangle$, $\Sigma_{vv,I}=\langle \left(\delta v_{I}\left(t\right)\right)\left(\delta v_{I}\left(t\right)\right)\rangle$, $\Sigma_{vv}=\Sigma_{vv,h}+\Sigma_{vv,I}$, and similarly for $\Sigma_{vx}$.
